# Multimodal text-audio sentiment in clinical aphasia speech using NLP

**DOI:** 10.3389/fdgth.2026.1740270

**Published:** 2026-08-10

**Authors:** Shamiha Binta Manir, Anai N. Kothari, William L. Gross, Priya Deshpande

**Affiliations:** 1 Department of Electrical and Computer Engineering, Marquette University, Milwaukee, WI, United States; 2 Department of Surgery, Medical College of Wisconsin, Milwaukee, WI, United States; 3 Department of Anesthesiology, Medical College of Wisconsin, Milwaukee, WI, United States

**Keywords:** acoustic features, affective tone, aphasia, clinical NLP, DistilBERT embeddings, multimodal speech analysis, sentiment analysis, weak supervision

## Abstract

**Introduction:**

Aphasia affects expressive and receptive communication and may influence the affective tone expressed during clinical speech tasks. This study presents an exploratory weakly supervised NLP analysis of positive/negative affective-tone proxies in AphasiaBank transcripts with paired audio.

**Methods:**

We extracted sentence embeddings from DistilBERT ($e\in R^{768}$) and recording-level acoustic summaries (${\mathrm{MFCC}}_{13}$, ZCR, RMS, spectral centroid, and spectral bandwidth; $a\in R^{17}$). Text and acoustic features were concatenated ($x=[e;a]\in R^{785}$) and classified using Random Forest models. Sentiment labels were generated using an SST-2-derived weak-supervision pipeline and should be interpreted as pseudo-labels rather than clinical ground truth. To evaluate modality contribution and potential leakage, we compared text-only, audio-only, and fused text-audio models under utterance-level and recording-disjoint splits. A small five-rater evaluation was used to examine human judgment alignment.

**Results:**

Under the recording-disjoint split, both the text-only and fused text-audio models achieved 97.9% accuracy and 0.791 macro-F1, while the audio-only model achieved 55.4% accuracy and 0.388 macro-F1. These results indicate that classification performance was primarily driven by textual embeddings, while the recording-level acoustic summaries did not improve performance over text-only features. Across the pseudo-labeled corpus, aphasic utterances were more often labeled negative than control utterances. Age-stratified summaries showed subgroup variation in pseudo-label distributions, but these patterns were treated descriptively because labels were model-derived. Human-rater agreement was low for aphasic utterances, indicating that affective-tone interpretation in fragmented clinical speech is ambiguous.

**Discussion:**

These findings should be interpreted as exploratory evidence about weakly supervised affective-tone proxies, not as validated clinical sentiment recognition. The results highlight both the promise of clinical NLP for aphasia discourse analysis and the need for independent human-labeled validation, utterance-aligned acoustic features, and careful control of domain, task, age, and topic bias.

## 1 Introduction

Aphasia, a multifaceted neurological disorder caused by impairment to the brain’s language centers, impairs an individual’s capacity for successful communication. Aphasia is generally induced by strokes, severe brain injuries, or neurological conditions such as tumors and infections, impacting various aspects of language processing, including speaking, comprehension, reading, and writing [1, 2]. Aphasia, characterized by a spectrum of symptoms including disjointed speech, word substitutions, and language comprehension deficits, poses significant challenges for individuals, impacting their communication capabilities, emotional health, and social interactions [3]. Aphasia does not affect intelligence; nonetheless, it substantially hinders the capacity to communicate and comprehend language, resulting in considerable challenges in everyday activities, mental well-being, and social interaction [4].

Speech-language therapy (SLT) is presently the primary treatment option for aphasia; qualified speech-language pathologists often personalize it to the specific deficits of the individual. This therapy aims to restore language abilities through structured tasks focused on word retrieval, sentence formation, and other communication methods such as gestures or written words [5]. Despite its widespread use, conventional speech-language therapy (SLT) does not consistently lead to recovery across all individuals with aphasia. Geographic accessibility, the presence of skilled professionals, and the financial burden of long-term care generally impose limitations [6, 7]. Moreover, SLT requires significant effort and time; hence, therapeutic outcomes can differ considerably across patients, typically influenced by the extent of the intervention [8, 9]. Notably, Wilson et al. conducted a longitudinal study demonstrating that recovery patterns are more strongly linked to lesion characteristics than to therapy intensity, suggesting that access to SLT is not the sole determinant of recovery outcomes [10]. This variability necessitates more accessible, scalable, and personalized treatment methodologies.

The rehabilitation process is significantly improved by family members, as success relies on consistent practice and support beyond clinical sessions [11]. Instructing caregivers in effective communication strategies—such as language simplification or the use of visual aids—fosters a supportive environment conducive to healing. Nonetheless, the emotional and cognitive burden on caregivers can be overwhelming, highlighting the necessity for tools designed to alleviate communication demands and enhance the quality of care provided. [12, 13]. Natural Language Processing (NLP) is a viable way to overcome the drawbacks of conventional aphasia treatment in light of these issues. We can investigate the linguistic and affective patterns in aphasic speech by using sophisticated computer models, therefore pointing up areas of difficulty and creating tools to help in communication. NLP’s capacity to examine speech inputs—especially fragmented or incomplete sentences common in aphasia—opens the way to automated systems able to help create intelligible speech forecasts or sentence complements in real time. For those with aphasia, these models could greatly improve communication not only in terms of expression of ideas and feelings but also in terms of dependency on in-person therapy sessions [14].

This work explores the AphasiaBank dataset using transformer-based NLP methods for weakly supervised affective-tone analysis of aphasic and control speech transcripts. Along with people with aphasia, this large and varied collection comprises transcripts from several linguistic activities including story retellings and conversational exchanges. Transformer-based models can help to solve the unusual syntax of aphasic speech so improving understanding of the communication problems this condition presents [15]. While sentiment analysis evaluates emotional expressiveness in both aphasic and control groups, topic modeling seeks for repeating trends in aphasic speech [16].

Beyond characterizing linguistic and affective-tone patterns in aphasia, this work is intended to support future development of clinically interpretable NLP tools for aphasia discourse analysis [17–19]. By identifying model-derived linguistic and affective-tone proxy patterns, such systems may eventually help researchers and clinicians better understand communication challenges in aphasia. However, the present study is exploratory and does not propose a diagnostic instrument or a validated treatment system.

This study examines whether combining linguistic text embeddings with recording-level acoustic summaries provides additional information for weakly supervised affective-tone classification in aphasic speech. Additionally, we investigate whether transformer-derived embeddings in more straightforward models (like Random Forests) can match or enhance transformer-based classifiers, especially when adjusted for demographic subgroups like gender and age.

Although our tasks (e.g., picture description, story retell) do not explicitly induce emotion, spontaneous utterances in these settings routinely convey *affective tone* (e.g., frustration, relief, self-evaluation, meta-comments). We therefore analyze affective tone rather than elicited emotion.

We employ a dual-model framework to assess the advantages and disadvantages of both deep and standard learning methods. We begin by employing a pretrained sentiment analysis pipeline to assign binary sentiment labels to utterances. The tagged samples are further utilized to refine transformer models (DistilBERT), categorized by age. We extract contextualized sentence embeddings from each fine-tuned model. These embeddings fulfill two functions: (1) they are utilized for direct sentiment classification employing transformer-based models, and (2) they are provided to Random Forest classifiers as fixed-length input representations.

This methodology enables a comparison of transformer-based classification with traditional models for performance and interpretability, particularly in clinical or resource-limited settings. Furthermore, we enhance the study by using acoustic features (e.g., MFCC, ZCR, RMS) alongside text embeddings for multimodal sentiment classification. This pipeline evaluates whether multimodal integration provides measurable improvement over text-only representations while accounting for demographic subgroup patterns.

## 2 Literature review

### 2.1 Sentiment analysis in aphasia

The integration of NLP into healthcare has led to breakthroughs in areas such as clinical text mining, sentiment analysis, and language disorder analysis. Transformer models, including BERT and RoBERTa, have been successfully applied to clinical text, helping analyze patient emotions and symptoms [16]. These models are particularly effective in handling fragmented or incomplete data, making them suitable for aphasia research, where speech is often disrupted [17]. Recent work in multilingual NLP demonstrates how sentiment analysis can uncover emotional markers within fragmented clinical speech, offering insights into cognitive and emotional states [18].

Topic modeling methods, especially LDA and BERTopic, have become widely used for analyzing healthcare text data, revealing thematic structures within patient speech [19]. LDA has been applied to large clinical datasets, providing insights into linguistic patterns and thematic reductions common in aphasic speech [20]. In particular, BERTopic has shown superior performance in clustering fragmented speech data, making it useful for extracting meaningful themes in aphasia-related studies [21].

Aphasia research has increasingly turned to computational methods for language processing, incorporating NLP techniques to analyze fragmented and incomplete speech. Topic modeling enables the extraction of recurring themes and linguistic deficits in aphasic discourse, revealing reduced narrative complexity and reliance on generic terms [22]. These models have been adapted to handle fragmented syntax and language errors, highlighting the need for specialized transformer models to improve sentiment analysis and topic extraction in aphasia [23]. To assess emotional responses in aphasic patients during clinical interviews, Ortiz-Perez et al. created a deep learning-based pipeline combining speaker identification, transcription, and facial emotion recognition, so demonstrating high degrees of frustration and neutral affect when patients struggle to understand speech [24].

### 2.2 NLP for clinical applications

Transformer models such as BERT have been adapted to clinical applications, focusing on sentiment analysis and the extraction of linguistic patterns from fragmented speech data. These models have been successfully fine-tuned for handling low-resource clinical datasets, a challenge often present in aphasia research [25]. Studies emphasize that transformers, by identifying emotional cues and linguistic markers, can offer insights into the emotional and cognitive states of patients with neurological impairments [26]. Fine-tuning these models for fragmented speech allows for more accurate analyses, critical for understanding the emotional and communicative deficits in aphasia. Our own recent work also demonstrates the utility of large language models for aphasia-related tasks, including question answering and text prediction [27].

The use of NLP in cognitive neuroscience has expanded, focusing on the relationship between language impairments and cognitive disorders such as aphasia [28]. Computational models like BERT have been employed to analyze speech patterns, helping researchers understand the effects of brain damage on language production [29]. These models have contributed to research on neuroplasticity and language recovery, offering new avenues for therapeutic interventions in aphasia [30].

Research in sentiment analysis for speech impairments has grown, using deep learning models to identify emotional states in fragmented speech [31]. Models like BERT have been adapted to assess the emotional impact of aphasia, revealing that individuals with the disorder often display heightened negative sentiment due to frustration and communication difficulties [32]. By refining these models to handle error-prone speech data, researchers have improved their ability to capture emotional variations, aiding clinicians in developing more targeted emotional therapies for aphasic patients [33].

Despite the advancements, significant challenges remain in adapting NLP models to handle clinical language data, particularly in cases of fragmented or incomplete speech. However, recent studies demonstrate the effectiveness of combining ProdLDA with transformer-based embeddings to enhance the interpretability of thematic distributions, offering a powerful tool for analyzing aphasia [34]. This combination allows for more precise modeling of linguistic impairments, highlighting the opportunities for developing automated therapeutic tools to complement existing interventions [35].

### 2.3 Multimodal approaches in speech analysis

The pilot study conducted by Purdy and Van Dyke on Multimodal Communicative Training (MCT) shows that teaching people with aphasia both verbal and nonverbal skills (e.g., gesture, drawing, writing, and pointing) at the same time can help them switch between modalities and communicate more effectively, particularly if their semantic representations are still intact [36]. The ability to interpret language-related neural activity holds revolutionary potential for clinical natural language processing, age-aware models, and sentiment analysis in aphasia, especially as integrated approaches across text, speech, image, and video modalities reveal deeper insights into cognitive flexibility and communication strategies in neurodiverse populations. This is made possible by advancements in artificial intelligence and multimodal brain decoding [37]. Understanding that those with aphasia sometimes rely on gestures to augment limited speech, Lee et al. presents a graph-based multimodal model learning the intricate interactions between spoken language and co-speech gestures. By concentrating on disfluency-aware speech traits and gesture patterns, the method not only improves the diagnosis value of gestures—often more informative than auditory clues alone—but also improves classification of aphasia kinds. This work offers doctors a more quick and easy tool to assist customized treatments for people with aphasia [38]. Privitera et al. (2024) examine the impact of artificial intelligence, including deep learning and natural language processing, on the diagnosis and treatment of aphasia. They emphasize significant advantages while also expressing concerns over bias, ethical implications, and insufficient linguistic diversity in existing models [13]. Kim et al. presented a gesture-aware zero-shot ASR framework using multimodal large language models to combine iconic gestures with disfluent speech, so greatly enhancing transcription accuracy and interpretive depth for people with aphasia, beyond audio-visual speech recognition [39]. Building on the significance of nonverbal cues in aphasia, Lee et al. developed a multimodal model combining gesture and speech patterns to automatically identify aphasia subtypes—finding that gestures, more than acoustic features, expose key variations between types like Broca’s and Wernicke’s [38].

### 2.4 Age-based NLP models for sentiment analysis

Demmelmaier et al. show in their thesis the effectiveness of Natural Language Processing (NLP) in estimating an author’s gender based on linguistic patterns, therefore obtaining an accuracy rate of 82%. This result emphasizes how well NLP methods might be used in textual data demographic analysis [40]. Particularly in zero-shot environments, Krugmann et al. show that large language models (LLMs) such as GPT-4 and Llama 2 may match or even surpass conventional transfer learning models in sentiment analysis, therefore implying a change toward more accessible, prompt-based methodologies in marketing research [41]. Díaz et al. show that many popular sentiment analysis tools tend to interpret language about older adults more negatively than the same language about younger people; just because of the words used. Their work is a powerful reminder that algorithms can quietly reflect and reinforce social biases, especially when built on data that doesn’t represent everyone [42]. A study predicted user age based on tweets by means of sentiment analysis and machine learning. Focusing on elders, adults, and teenagers, they demonstrated how a person’s age group may be reflected in text’s emotional expression and writing style [43].

## 3 Methodology

**Figure 1:**
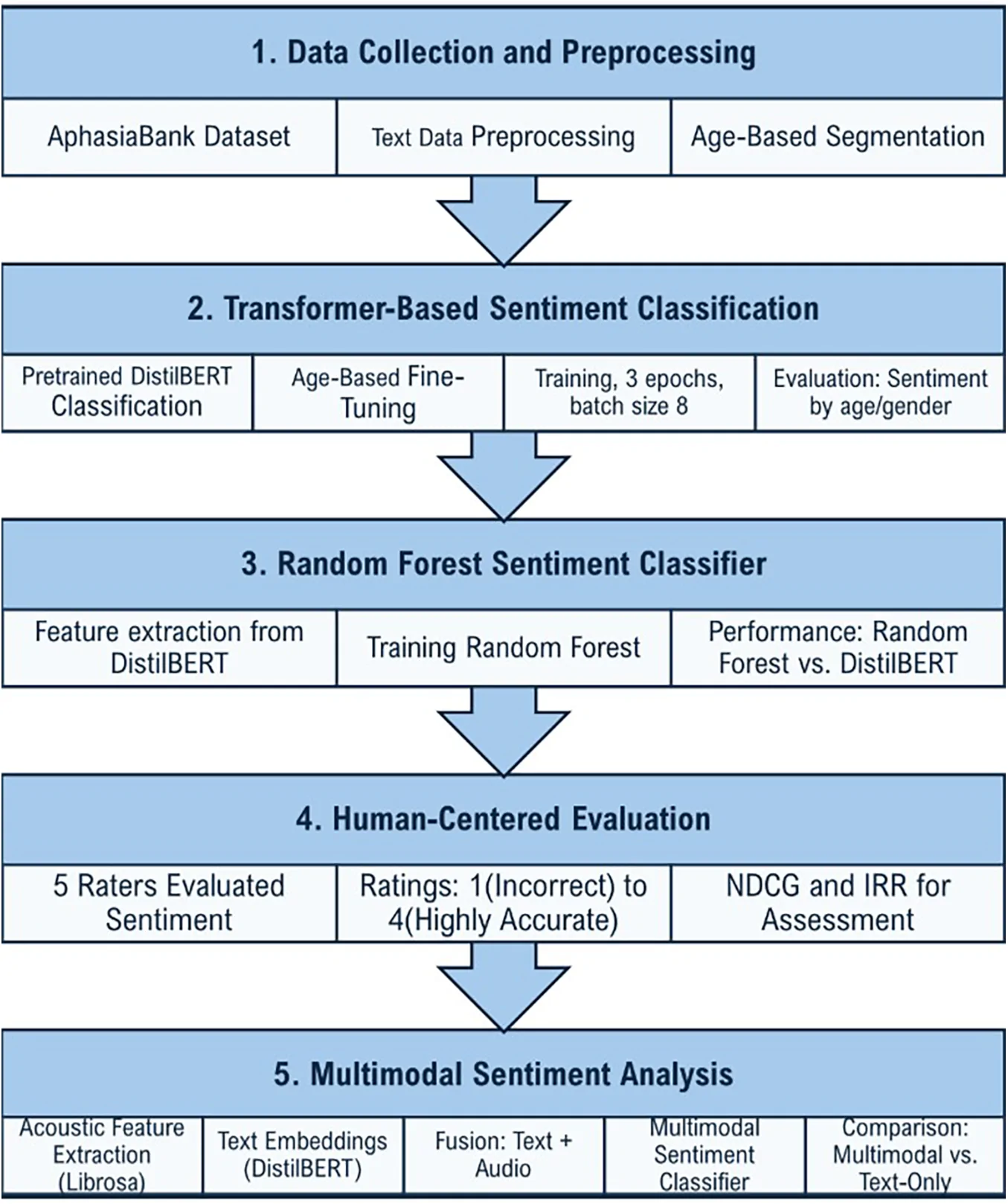
Overview of the multimodal sentiment analysis pipeline.

The overall workflow of the proposed weakly supervised multimodal sentiment analysis pipeline is shown in Figure 1.

### 3.1 Data collection and preprocessing

#### 3.1.1 AphasiaBank dataset overview

This study utilized the AphasiaBank dataset, a comprehensive collection of audio recordings and transcriptions documenting interactions involving individuals with aphasia and control participants [44]. The dataset, provided in CHAT format, includes both verbal speech and non-verbal cues such as gestures, which posed preprocessing challenges for natural language processing (NLP) tasks. To address these challenges, extensive preprocessing was performed to prepare the data for sentiment analysis and multimodal integration.

#### 3.1.2 Text data preprocessing

The preprocessing began with extracting participant utterances marked with the *PAR: identifier in the CHAT files. Python scripts were employed to recursively process the dataset, ensuring all relevant files were analyzed. Non-verbal cues, such as annotations for laughter or gestures (e.g., “laughs,” “sighs”), were removed alongside special characters and incomplete sentences. Sentences with fewer than three words were discarded to ensure the retention of meaningful linguistic content. The cleaned transcriptions were aligned with participant demographic data (age, gender) and task metadata (e.g., task types like storytelling or conversation), enabling enriched contextual analyses. After preprocessing, two distinct datasets were created: one for aphasic speech and another for non-aphasic control speech. These datasets were converted into structured formats, such as CSV files, to facilitate downstream tasks including sentiment analysis and multimodal classification. The sentiment labeling pipeline uses the cleaned utterances as input, and the resulting transformer models are fine-tuned based on the user’s age.

#### 3.1.3 Sentiment labeling strategy

We employed a pretrained sentiment classifier derived from the Stanford Sentiment Treebank/SST-2 sentiment benchmark (implemented through Hugging Face’s pipeline) to assign binary sentiment pseudo-labels (positive or negative) to each utterance [45]. These pseudo-labels offered scalable annotations for training, eliminating the need for human labeling of the entire dataset. Although computerized labeling generates certain noise, its consistency facilitates large-scale analysis of aphasic speech. A human-centered evaluation was subsequently performed to evaluate the congruence between machine predictions and human perception. Because SST-2 is not a clinical aphasia dataset, these labels were treated as weak pseudo-labels for exploratory modeling rather than as ground-truth clinical sentiment annotations.This framing is consistent with weak-supervision approaches, where model- or rule-derived labels can support scalable exploratory modeling but require cautious interpretation when independent human ground truth is unavailable [46]. Because pretrained language models and sentiment classifiers can be sensitive to domain mismatch, especially when applied outside their original training distribution, the SST-2-derived labels were interpreted as affective-tone proxies rather than validated clinical sentiment labels [47, 48].

#### 3.1.4 Age-based segmentation and dataset splitting

We investigated how age affected emotional expression in aphasic speech. The age groups were Young (<40), Middle-Aged (40–59), and Elderly (60+). pandas.cut() allowed you to assign participants to categories depending on their age metadata, therefore facilitating these age bins. This part allowed one to train and test models considering age.

Following their labeling, the datasets were filtered to retain just samples with appropriate sentiment annotations—0 for negative and 1 for positive. Every group then was split into sequences of no more than 512 tokens using the DistilBERT tokenizer. Each age group could undertake their own fine-tuning using Hugging Face’s Trainer API and consistent training schedule (3 epochs, batch size of 8 evaluations at each epoch). For every group, stratified sample divides data into 80% training and 20% testing data. This maintained the opinion balance.

Sentence-level embeddings were extracted from every age-specific DistilBERT model once the [CLS] token’s final hidden state had been optimised. These embeddings were maintained for subsequent initiatives, including developing a Random Forest classifier to evaluate age-specific text representations. This dual-model approach helped us to test both transformer-based and tree-based sorting techniques for demographic groups. Additionally investigated using processed embeddings was the effect of linguistic variations linked to age on sentiment polarity.

### 3.2 Transformer-based sentiment classification

#### 3.2.1 Pretrained DistilBERT sentiment classification

This study employed DistilBERT, a distilled lightweight transformer-based model, to represent utterances for downstream positive/negative pseudo-label classification [49]. DistilBERT was chosen for its computational efficiency and ability to process fragmented and irregular syntax common in aphasic speech. The dataset for this analysis was divided into aphasic and control groups, with the aphasic dataset consisting of 73,585 sentences and the control dataset comprising 69,467 sentences. Preprocessing ensured that the datasets were balanced and free from noise, enabling accurate sentiment predictions.

#### 3.2.2 Age-based DistilBERT training and fineTuning

We tuned separate DistilBERT models utilizing the pseudo labeled data for three demographic groups: Young, Middle Aged, and Elderly, in order to examine age-related changes in sentiment perception and linguistic patterns among individuals with aphasia. To preserve the sentiment distribution and minimize demographic confounds, each model was trained individually using stratified data specific to its corresponding group.

This difference between training and validation loss points to the models starting to overfit after the first epoch even if they effectively learnt age-specific traits. In older populations especially, where linguistic output may be more fractured or emotionally dubious, this is especially noteworthy since it complicates the classification process. These findings underline the need of applying regularizing techniques, such early stopping or dropout corrections, to avoid overfitting when fine-tuning models on age-sensitive speech data.

The [CLS] token embeddings from each sentence were extracted and preserved for two subsequent tasks: direct classification employing transformer models, and fixed-input classification through Random Forests to evaluate the effectiveness of simpler classifiers in leveraging deep contextual embeddings. Later applications for these embeddings included multimodal fusion and conventional machine learning comparisons in downstream classification challenges. Customizing sentiment models at the demographic level helped the system to more precisely capture emotional expression patterns in aphasic speech and improve its adaptation to speaker variability.

### 3.3 Random forest sentiment classifier

To supplement the transformer-based analysis, we trained Random Forest classifiers using DistilBERT-derived sentence embeddings and acoustic features. Rather than treating this comparison as a direct architectural comparison between transformers and Random Forests, the revised analysis evaluates how different feature sets perform under the same classifier: text-only, audio-only, and fused text-audio representations.

Let $e\in R^{768}$ denote the DistilBERT sentence embedding and $a\in R^{17}$ denote the standardized acoustic vector. We form the fused representation $x=[e;a]\in R^{785}$ and train a Random Forest f(⋅) to estimate $\hat{y}=f(x)$ with y∈0,1, where 0 indicates negative and 1 indicates positive pseudo-labels. Because the acoustic features were extracted as recording-level summaries, multiple utterances from the same recording can share identical acoustic vectors. To address this issue, we report both an utterance-level split and a recording-disjoint split based on acoustic-vector grouping. The recording-disjoint split is used as the more conservative evaluation because it prevents identical recording-level acoustic summaries from appearing in both training and test sets.

#### 3.3.1 DistilBERT embedding extraction

We extracted high-dimensional sentence embeddings from the age-specific DistilBERT models fine-tuned in the previous stage to help the usage of conventional machine learning models. We specifically used the last hidden state of the [CLS] token, which codes a global view of the input sentence. For every phrase in the Young, Middle-Aged, and Elderly age ranges, these embeddings were calculated and then kept as NumPy arrays. With non-transformer-based models, this stage allowed a consistent, fixed-length representation of linguistic characteristics easily usable for downstream categorization.

#### 3.3.2 Classifier training and evaluation pipeline

Subsequently, Random Forest classifiers were trained utilizing the resulting embeddings, so establishing a tree-based baseline for sentiment categorization. Stratified datasets that preserve class balance during the training and testing division were generated by consolidating embeddings from each age group with corresponding binary sentiment labels (0 for negative and 1 for positive). The Random Forest model was trained using an 80/20 train-test ratio to provide fair assessment across demographic groups. This method allows us to examine if the sentiment classification efficacy of smaller, interpretable models aligns with that of transformer-based architectures.

### 3.4 Human-centered evaluation of sentiment predictions

#### 3.4.1 Manual evaluation using IRR and NDCG

Following the standard discounted cumulative gain framework for ranked relevance evaluation, for a list of n items with relevance scores $r_{j}\in 1,2,3,4$, the DCG of a rater is [50]

$$\mathrm{DCG}=\sum_{\;j=1}^{n}\frac{2^{r_{j}}-1}{\log_{2}\;(j+1)}.$$

We form a consensus score per item by averaging across raters and define the ideal DCG (IDCG) by sorting items by consensus. We then compute

$$\mathrm{NDCG}=\frac{\mathrm{DCG}}{\mathrm{IDCG}}.$$

Because NDCG is computed as the ratio of DCG to IDCG, values are bounded between 0 and 1, with higher values indicating stronger alignment between an individual rater’s ranking and the consensus ranking.

With N items, n raters, and k categories, let $n_{ij}$ be the number of raters who assign item i to category j. Define

$$P_{i}=\frac{1}{n(n-1)}\sum_{\;j=1}^{k}n_{ij}(n_{ij}-1),\;\bar{P}=\frac{1}{N}\sum_{i=1}^{N}P_{i},\;P_{e}=\sum_{\;j=1}^{k}{\left(\frac{1}{Nn}\sum_{i=1}^{N}n_{ij}\right)}^{2},$$

then Fleiss’ $\kappa =(\bar{P}-P_{e})/(1-P_{e})$ [51]. To assess the model’s success in capturing sentiment-particularly within the intricate, fragmented linguistic patterns of aphasia-we performed a manual evaluation with the assistance of five independent raters. The evaluators assessed a varied collection of sentences from three categories: aphasic participants, non-aphasic controls, and DistilBERT-generated predictions.

Each assessor was instructed to rate the sentiment on a 1–4 scale based on fit between predicted emotion and the utterance’s tone, conversational context, expressed intensity, and coherence given any language disruptions.

The language samples assessed by the evaluators encompassed various scenarios-from medically pertinent assertions to informal conversations-and covered diverse age and gender demographics.

Because affective-tone judgment in fragmented aphasic speech is subjective, this evaluation was designed as a small feasibility audit rather than as definitive validation of model correctness. Inter-rater agreement was later examined using Fleiss’ κ, and ranking-level agreement was examined using NDCG.

Apart from the raw scores, we computed Normalized Discounted Cumulative Gain (NDCG) for the Predictions, Aphasia, and Control subsets. NDCG was used as a ranking-alignment measure between individual raters and the consensus ranking, not as a direct measure of classification accuracy. Because the evaluation set was small and inter-rater agreement was low for aphasic utterances, this analysis was treated as a feasibility audit rather than definitive validation of model correctness. This human-centered evaluation extends our prior aphasia-related NLP work by adding a small rater-based assessment of perceived affective-tone alignment [27].

### 3.5 Multimodal sentiment analysis

This study presents a multimodal sentiment analysis framework that integrates textual embeddings from DistilBERT with acoustic features extracted using Librosa. The primary objective is to classify sentiment polarity (positive/negative) by leveraging both linguistic and prosodic features, allowing for a deeper analysis of emotional expression in speech. The pipeline is designed for computational efficiency through batch processing and GPU acceleration, ensuring scalability for large datasets. Acoustic features were extracted using Librosa following embedding extraction from DistilBERT. The two feature sets were then merged into a unified 785-dimensional vector for sentiment classification using a Random Forest classifier. The leakage-aware multimodal ablation pipeline is summarized in Algorithm 1.

Algorithm 1 **Leakage-Aware Multimodal Ablation Pipeline.**

1: D← directory containing paired CHAT transcripts and audio files

2: F*chat,F*audio← list of matched transcript and audio files

3: S←∅          ⊳ Initialize multimodal sample set

4: Load DistilBERT tokenizer and embedding model

5: Load SST-2-derived sentiment pipeline for weak pseudo-labeling

6: Initialize StandardScaler for acoustic feature normalization

7: **for** each transcript file $f_{\mathrm{cha}}\in F*\mathrm{chat}$ **do**

8:  f*audio←corresponding audio file for $f_{cha}$

9:  **if** $f_{audio}$ exists **then**

10:   $T\leftarrow ExtractUtterances(f_{cha})$       ▹ Cleaned utterances and metadata

11:   $A\leftarrow ExtractRecordingAcoustics(f_{audio})$      ▹ Recording-level 17-D acoustic vector

12:   **if** T≠∅ and A≠∅ **then**

13:    a←Normalize(A)

14:    **for** each utterance $t_{i}\in T$ **do**

15:     $y_{i}\leftarrow SST2PseudoLabel(t_{i})$          ⊳ 0= negative, 1= positive

16:     $e_{i}\leftarrow DistilBERTEmbedding(t_{i})$  ⊳ 768-D text embedding

17:     $x_{i}^{text}\leftarrow e_{i}$

18:     $x_{i}^{audio}\leftarrow a$

19:     $x_{i}^{\;fused}\leftarrow [e_{i}∥a]$  ▹ 785-D fused vector

20:     $g_{i}\leftarrow Hash(a)$          ▹ Recording-level acoustic-vector group

21:     $S\leftarrow S\cup (x_{i}^{\mathrm{text}},x_{i}^{\mathrm{audio}},x_{i}^{\mathrm{fused}},y_{i},g_{i})$

22:    **end** **for**

23:   **end** **if**

24:  **end** **if**

25: **end** **for**

26: $X^{\mathrm{text}},X^{\mathrm{audio}},X^{\mathrm{fused}},Y,G\leftarrow \mathrm{Unpack}(S)$

27: $\left(I^{u}*\mathrm{train},I^{u}*\mathrm{test})\leftarrow \mathrm{UtteranceLevelSplit}(Y\right)$

28: $\left(I^{r}*\mathrm{train},I^{r}*\mathrm{test})\leftarrow \mathrm{RecordingDisjointSplit}(Y,G\right)$

29: **for** each feature set **do**

30:  Train Random Forest on $X[I^{u}*train],Y[I^{u}*train]$

31:  Evaluate on $X[I^{u}*test],Y[I^{u}*test]$

32:  Train Random Forest on $X[I^{r}*train],Y[I^{r}*train]$

33:  Evaluate on X [Ir*test], Y [Ir*test]

34: **end** **for**

35: **return** classification reports for text-only, audio-only, and fused models under both split settings

To reduce the possibility of leakage from repeated recording-level acoustic summaries, the analysis reports both an utterance-level split and a recording-disjoint split based on acoustic-vector grouping. The recording-disjoint split is treated as the more conservative evaluation setting.

#### 3.5.1 Extracting text embeddings using DistilBERT

For textual processing, DistilBERT was used to generate sentence-level embeddings from the cleaned utterances. Each utterance was tokenized and processed in batches to optimize memory usage. The final hidden state of the [CLS] token was used as a 768-dimensional embedding representation. These embeddings were then used as text-only features and were also concatenated with recording-level acoustic summaries for the fused text-audio representation.

#### 3.5.2 Acoustic feature extraction using librosa

In parallel, Librosa was used to derive acoustic features that capture non-linguistic speech characteristics relevant to prosodic and acoustic analysis [52]. The extracted features include: (i) Mel-Frequency Cepstral Coefficients (MFCCs); (ii) Zero Crossing Rate (ZCR); (iii) Root Mean Square (RMS) Energy; and (iv) Spectral Centroid and Bandwidth. Each recording was represented by a 17-dimensional acoustic summary consisting of 13 MFCC means, RMS energy, zero-crossing rate, spectral centroid, and spectral bandwidth. These acoustic summaries were computed at the recording level and then attached to each extracted utterance from the corresponding recording.

#### 3.5.3 Combining text and audio features

Once feature extraction is complete, the normalized acoustic features and textual embeddings are combined to generate a single feature representation for each utterance. DistilBERT produces sentence-level embeddings (768-dimensional vectors) derived from the final hidden state of the [CLS] token. Simultaneously employing StandardScaler, acoustic features—including MFCCs (13), RMS, ZCR, spectral centroid, and spectral bandwidth (totaling 17 features)—are standardized to ensure consistency across feature types. For each phrase (768 text plus 17 audio), the resulting vectors are concatenated into a final 785-dimensional feature vector, thus providing a comprehensive multimodal representation of the emotional content.

#### 3.5.4 Multimodal sentiment classifier: training

A Random Forest classifier is then trained to predict sentiment labels (*positive* or *negative*). The training dataset undergoes stratified splitting to ensure balanced representation of both sentiment classes in the training and test sets, mitigating biases that may arise due to class imbalance.

## 4 Experiments and results

### 4.1 Exploratory analysis

**Figure 2:**
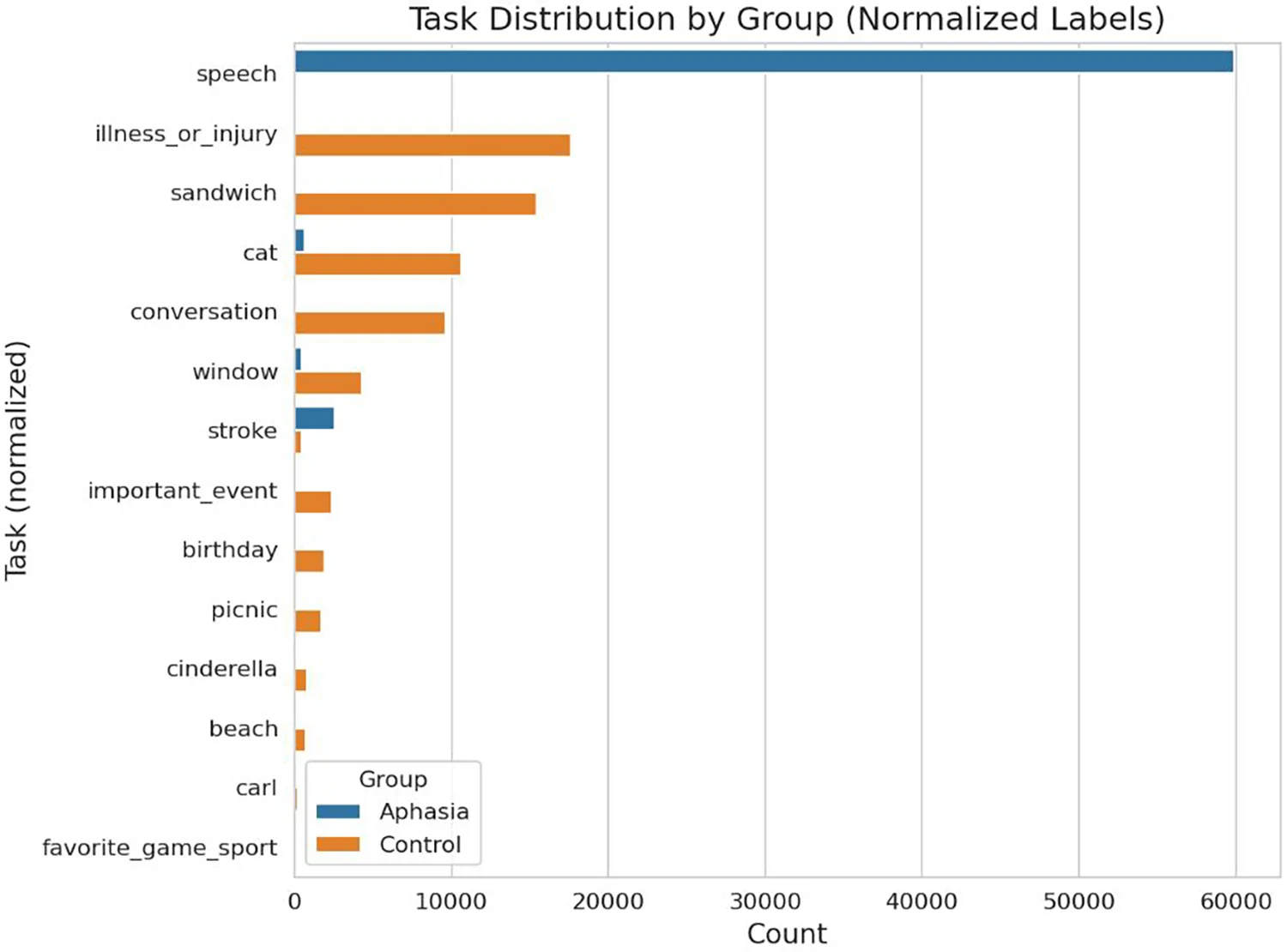
Task distribution by group (after label normalization). Task labels were lowercased and harmonized (e.g., Speech → speech, Illness or Injury → illness_or_injury) prior to aggregation. Counts are shown by group to document sampling differences across tasks that could confound downstream statistics. This normalization step is used for all subsequent analyses.

**Figure 3:**
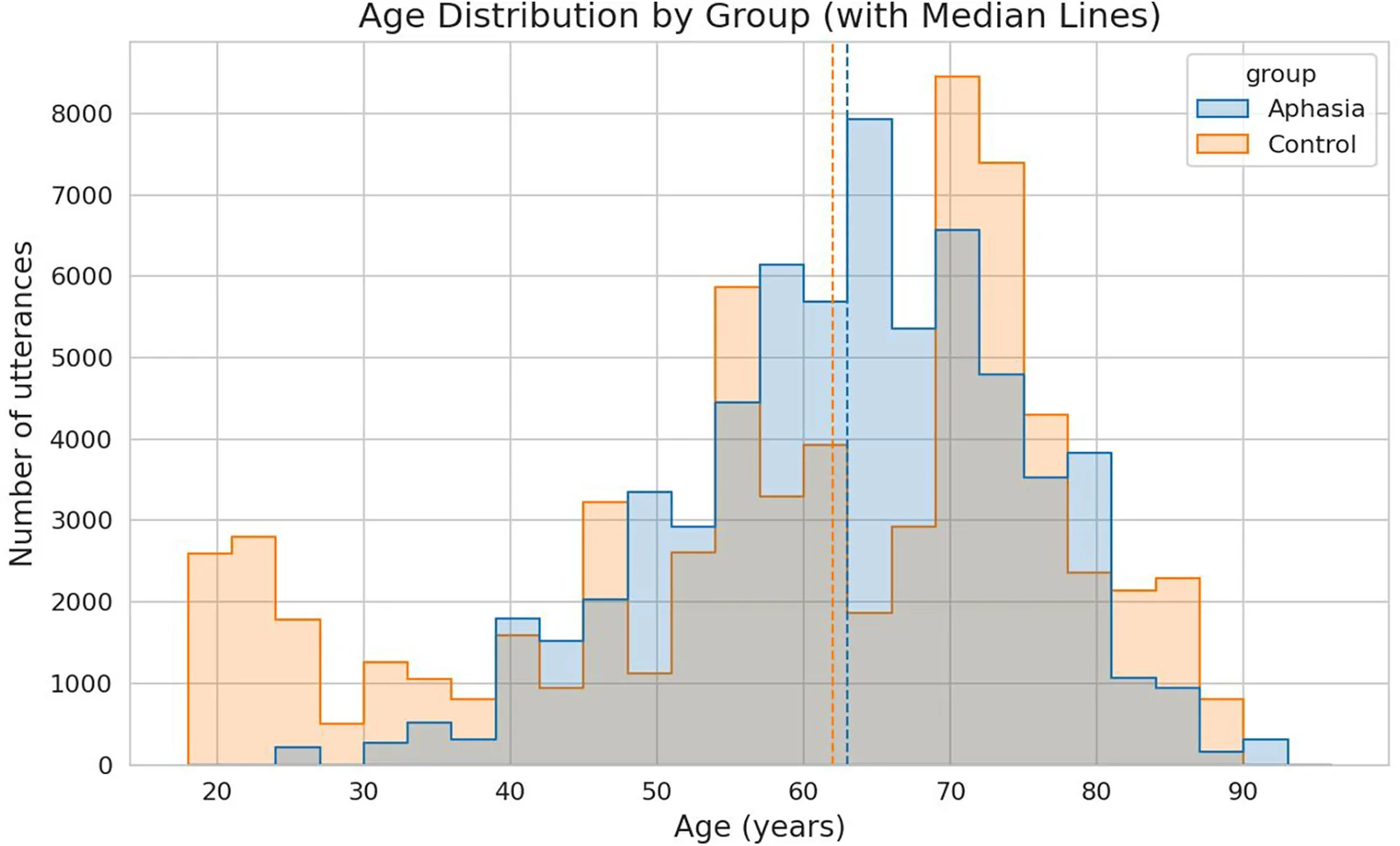
Age distribution by group with medians. Overlaid histograms show the utterance-level age distribution for aphasic and control groups, with dashed vertical lines marking group medians. This visualization documents age imbalance that may confound sentiment patterns. Model evaluations are reported using utterance-level and recording-disjoint splits, with the recording-disjoint split treated as the more conservative setting.

**Figure 4:**
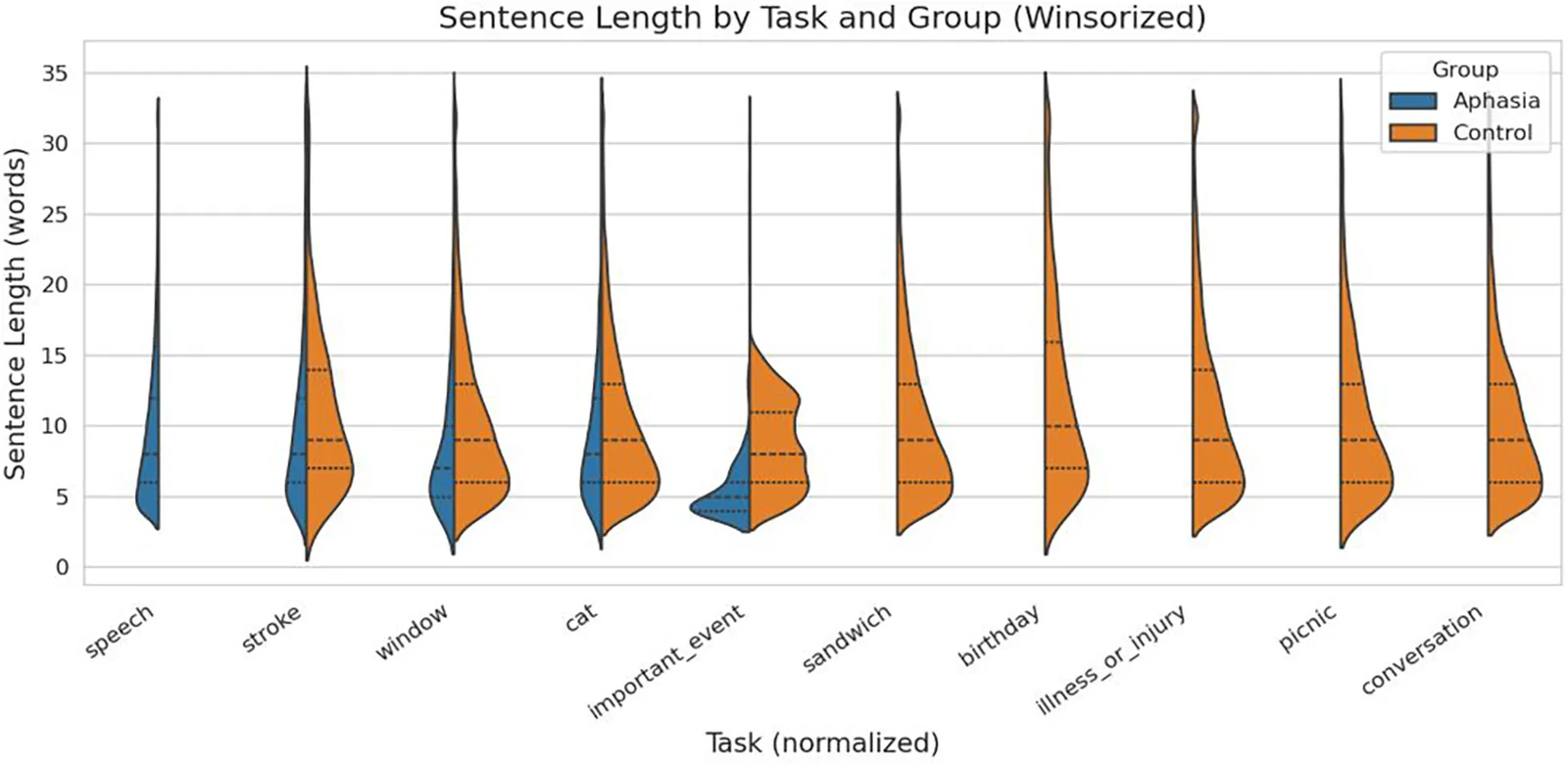
Sentence length by task and group (winsorized). Violin plots summarize per-utterance length (words) after 1%/99% winsorization to reduce leverage of outliers. Across tasks, aphasic utterances are shorter on average than controls, consistent with reduced fluency; task effects are retained to motivate task fixed effects in statistical models.

**Figure 5:**
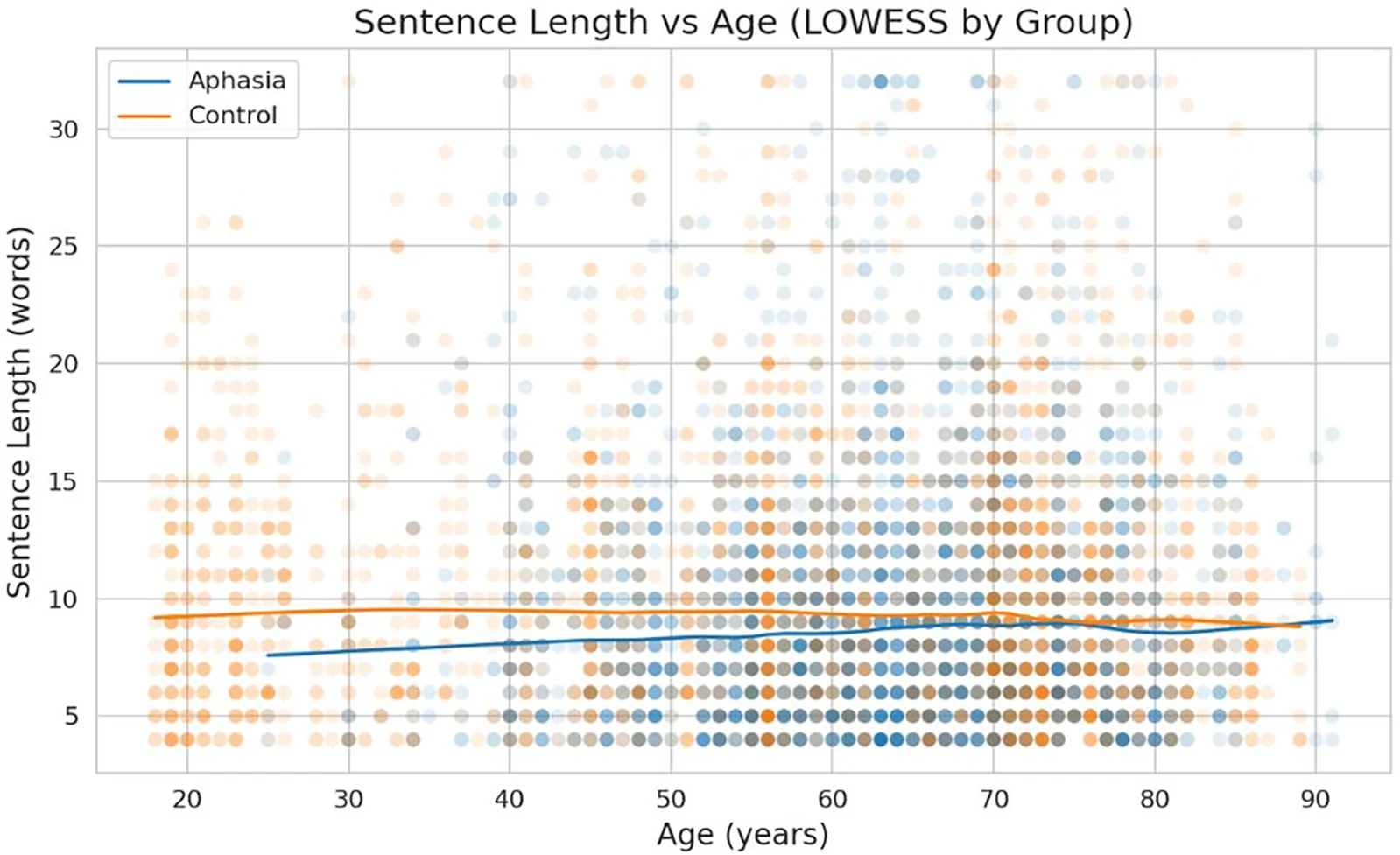
Sentence length vs. age with LOWESS trend by group. Scatter points (jittered) and group-wise LOWESS curves show weak age trends relative to the between-group difference. Analyses control for age either via stratified models or age covariates to ensure that group effects are not artifacts of cohort imbalance.

Exploratory task, age, and sentence-length distributions are shown in Figures 2–5 to document sampling patterns and potential cohort/task imbalance before downstream modeling.

### 4.2 Performance of transformer-based sentiment analysis

The weakly supervised pseudo-label analysis showed group-level differences in model-derived affective-tone distributions. After filtering to valid binary SST-2-derived pseudo-labels, the aphasia subset contained 26,446 utterances and the control subset contained 23,663 utterances. Aphasic utterances were more often assigned negative pseudo-labels (73.7%) than positive pseudo-labels (26.3%). Control utterances also showed more negative than positive pseudo-labels (59.3% vs. 40.7%), although the negative-label proportion was lower than in the aphasia group.

These results should be interpreted as model-derived affective-tone proxy patterns rather than direct evidence of emotional state. Because the labels were generated using an SST-2-derived weak-supervision pipeline, the observed group differences may reflect language structure, task content, topic, age distribution, or domain mismatch in addition to affective tone.

**Figure 6:**
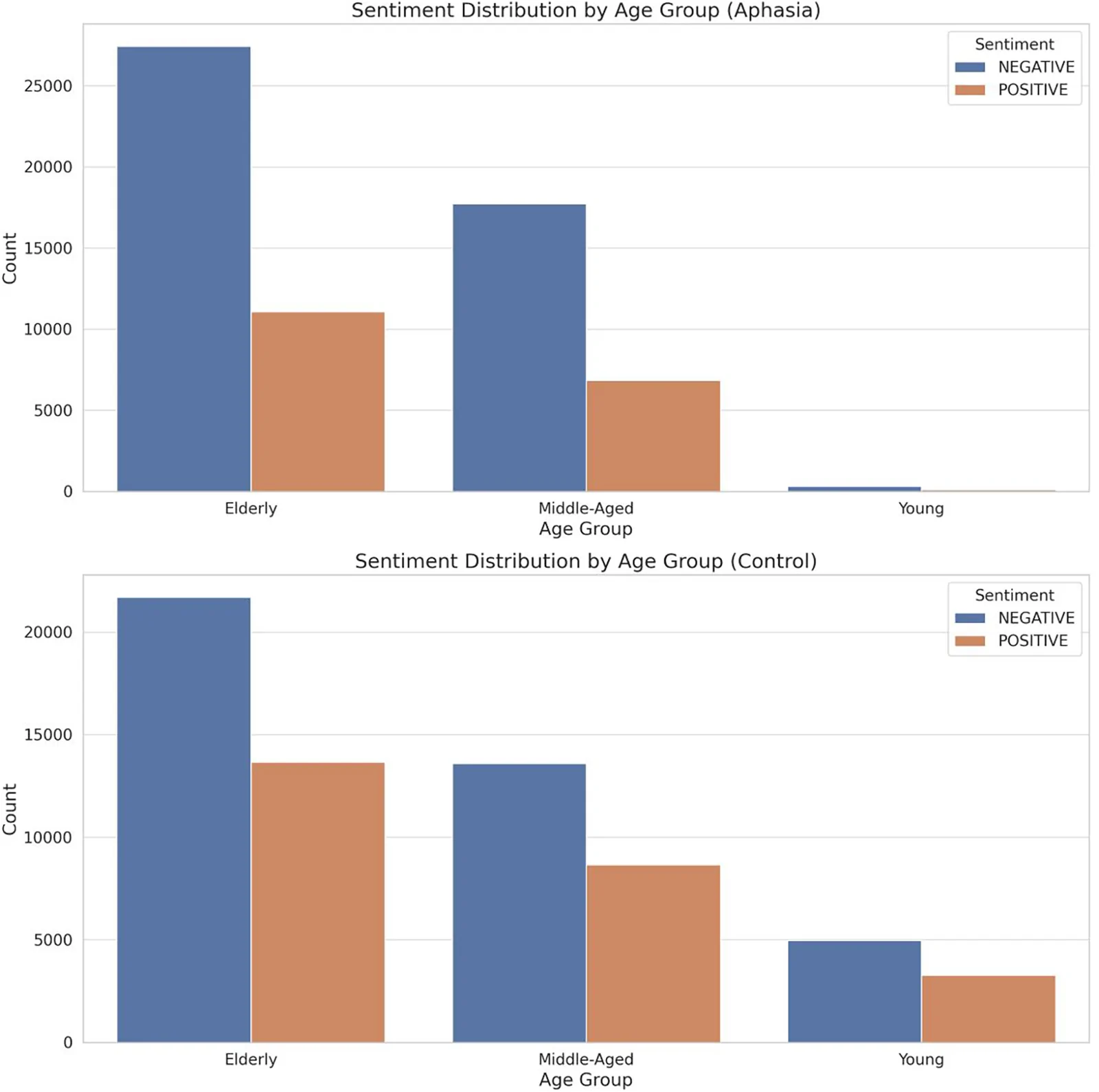
Distribution of SST-2-derived sentiment pseudo-labels by age group for aphasic and control utterances. Counts represent model-assigned affective-tone labels rather than independently validated emotional states. Age-related patterns are interpreted descriptively because pseudo-label distributions may reflect task, topic, sampling, or domain effects in addition to affective tone.

**Figure 7:**
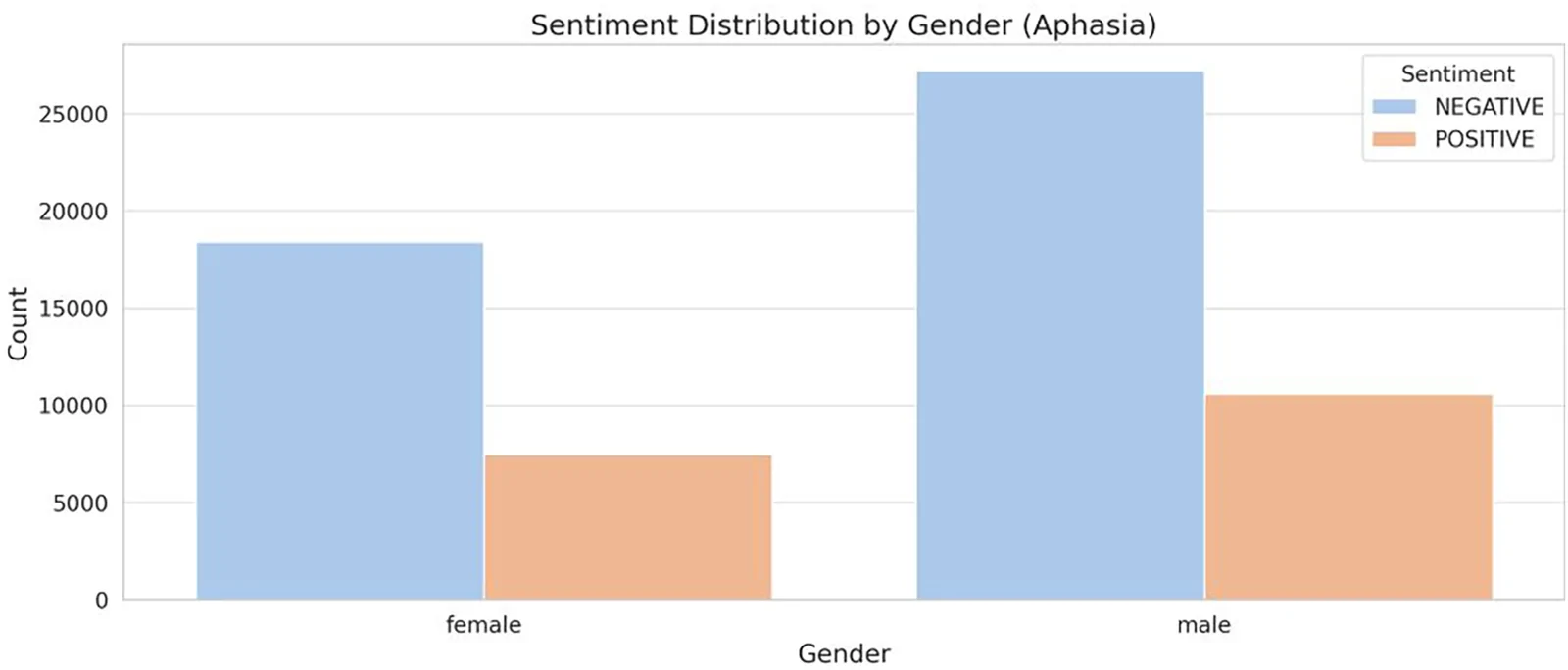
Distribution of SST-2-derived sentiment pseudo-labels by gender within the aphasia group. Male aphasic participants show a higher count of negative pseudo-labels in this corpus, but this pattern should be interpreted cautiously because labels are model-derived and may reflect sampling, task, or topic differences.

**Figure 8:**
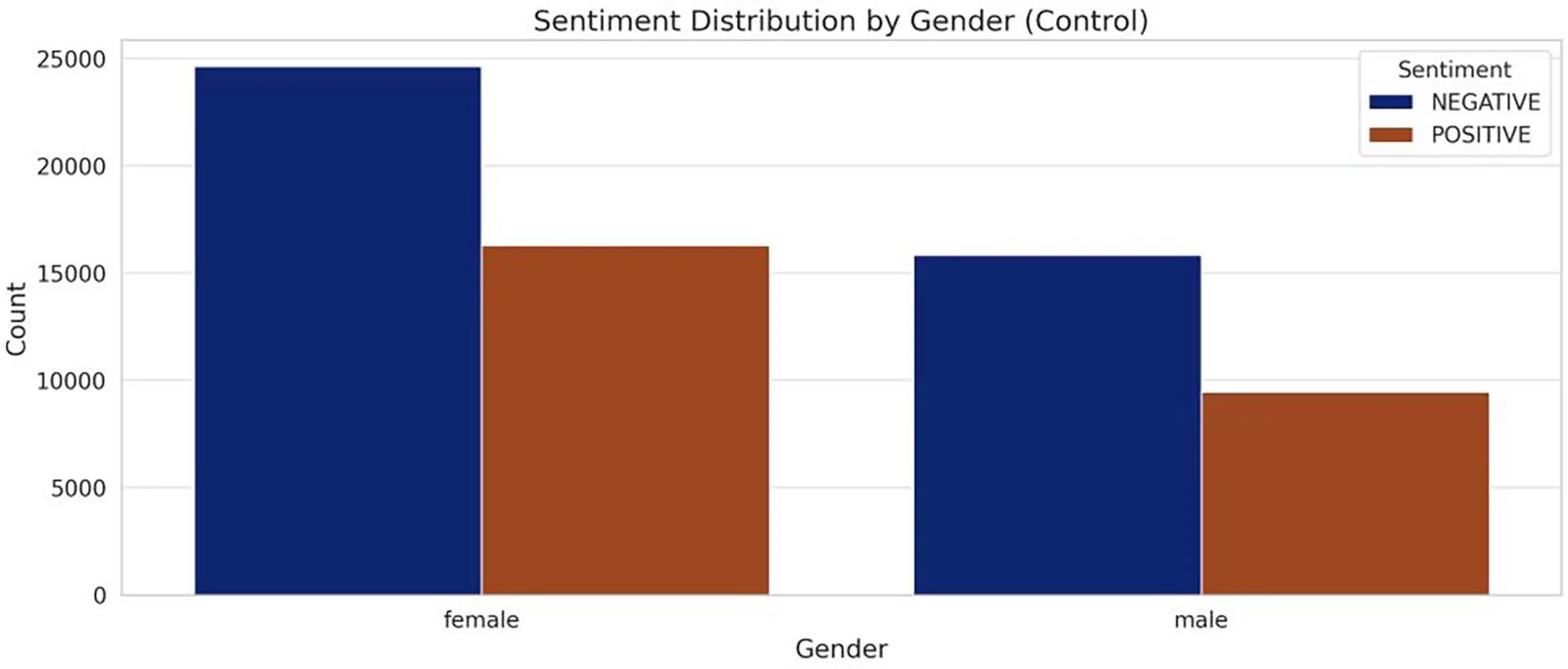
Distribution of SST-2-derived sentiment pseudo-labels by gender within the control group. The control utterances show a less negative-skewed pseudo-label distribution than the aphasia group, but these counts should be interpreted as model-derived affective-tone patterns rather than direct emotional measurements.

Age- and gender-stratified summaries were used as descriptive analyses to examine possible demographic patterns in the pseudo-label distributions. Age-stratified pseudo-label counts are shown in Figure 6, and gender-stratified pseudo-label distributions for aphasia and control groups are shown in Figures 7 and 8. These subgroup results are presented cautiously because they are based on pseudo-labels rather than independently validated clinical sentiment annotations.

#### 4.2.1 Baseline DistilBERT model on full dataset results

The baseline SST-2-derived pseudo-labeling pipeline was used to summarize broad affective-tone proxy patterns across aphasic and control utterances. As shown in Table 1, aphasic utterances had a higher negative pseudo-label proportion than control utterances. These results should be interpreted as weakly supervised affective-tone proxy patterns rather than clinically validated emotional states.

**Table 1:** SST-2-derived pseudo-label distribution in aphasic and control groups.

Group	Valid N	Positive pseudo-label	Negative pseudo-label
Aphasia	26,446	26.3	73.7
Control	23,663	40.7	59.3

Values are percentages and reflect weakly supervised affective-tone proxy labels rather than clinically validated emotional states.

#### 4.2.2 Age-based DistilBERT performance

Age-stratified pseudo-label summaries showed subgroup variation in model-assigned affective-tone labels. As summarized in Table 2, aphasic utterances showed higher negative pseudo-label proportions than control utterances across age groups. However, these subgroup patterns should be interpreted cautiously because the labels are SST-2-derived pseudo-labels and may reflect task, topic, sampling, age distribution, or domain mismatch rather than validated emotional differences.

**Table 2:** Age-stratified SST-2-derived pseudo-label distribution.

Age group	Group	Positive pseudo-label	Negative pseudo-label
Young (<40)	Aphasia	15.5	84.5
Young (<40)	Control	39.6	60.4
Middle-aged (40–59)	Aphasia	25.7	74.3
Middle-aged (40–59)	Control	41.3	58.7
Elderly (60+)	Aphasia	26.6	73.4
Elderly (60+)	Control	40.3	59.7

Values are percentages. These subgroup summaries are descriptive and should be interpreted cautiously because labels are model-derived pseudo-labels rather than clinical sentiment annotations.

The models had different learning patterns even with the same training setup: three epochs, an eight-batch size, and evaluation at every epoch. While the validation loss first dropped to 0.3126 but grew in later epochs, peaking at 0.4467, the training loss dropped gradually from 0.3423 to 0.0738 for the Middle-Aged group during three epochs. Training loss improved from 0.3398 to 0.0735 but validation loss rose from 0.3110 to 0.4905 during the same period in the Elderly group. Because validation loss increased in some age-specific models, these fine-tuning results were interpreted cautiously. The downstream classifier results are reported as agreement with SST-2-derived pseudo-labels, not as evidence that age-specific models captured validated emotional states.

#### 4.2.3 Model performance overview

Despite signs of overfitting in some age-specific DistilBERT fine-tuning runs, the extracted embeddings were useful for downstream pseudo-label classification. These results should be interpreted as model agreement with weak pseudo-labels rather than as validated sentiment-recognition performance. Therefore, the age-specific analyses are treated as exploratory and descriptive.

### 4.3 Performance of random forest classifier

To evaluate whether fixed text embeddings could support downstream pseudo-label classification, Random Forest classifiers were trained using DistilBERT-derived sentence embeddings. The age-specific Random Forest results are reported in Table 3. These metrics reflect agreement with SST-2-derived pseudo-labels rather than independently validated clinical sentiment accuracy.

**Table 3:** Age-specific Random Forest performance using DistilBERT text embeddings and SST-2-derived pseudo-labels.

Age group	Sentiment	Precision	Recall	F1-score	Support
Young	Negative (0)	0.90	0.99	0.94	212
Young	Positive (1)	0.83	0.38	0.53	39
Young	Accuracy	0.89			251
Middle-aged	Negative (0)	0.97	0.93	0.95	7,481
Middle-aged	Positive (1)	0.81	0.92	0.87	2,592
Middle-aged	Accuracy	0.93			10,073
Elderly	Negative (0)	0.96	0.95	0.95	10,841
Elderly	Positive (1)	0.86	0.89	0.87	3,929
Elderly	Accuracy	0.93			14,770

These metrics reflect agreement with weak pseudo-labels rather than independently validated clinical sentiment accuracy.

Across age groups, Random Forest classifiers showed high performance for the negative pseudo-label class but weaker performance for the positive class, especially in the Young aphasia subset. This pattern is consistent with class imbalance and should not be interpreted as evidence of true emotional-state detection. Instead, the results show that DistilBERT embeddings contain enough information for a simpler classifier to reproduce the weak pseudo-labeling patterns.

#### 4.3.1 Text-only, audio-only, and fused ablation analysis

To address potential information leakage and to isolate the contribution of each modality, we performed an additional ablation analysis comparing text-only, audio-only, and fused text-audio Random Forest classifiers on a common test set. Because the available acoustic features were summarized at the recording level, multiple utterances from the same recording shared identical acoustic vectors. Therefore, we report both an utterance-level split and a recording-disjoint split based on acoustic-vector grouping.

Under the recording-disjoint split, the text-only model achieved 97.9% accuracy and 0.791 macro-F1, while the fused text-audio model achieved the same performance. In contrast, the audio-only model achieved 55.4% accuracy and 0.388 macro-F1, with very low positive-class F1. These results indicate that the current classification performance is primarily driven by textual embeddings, while the recording-level acoustic summaries did not provide measurable improvement over text-only features. Accordingly, we interpret the multimodal component cautiously and treat the acoustic features as exploratory rather than as evidence of robust utterance-level affect detection.

#### 4.3.2 Effectiveness of combined features

The fused representation combined 768-dimensional DistilBERT embeddings with 17 recording-level acoustic features. However, the ablation analysis showed that fusion did not improve performance over text-only embeddings under the recording-disjoint split. This suggests that the current acoustic summaries did not add measurable predictive value beyond the textual features. Future work should use utterance-aligned acoustic features and affect-relevant prosodic descriptors such as pitch, loudness dynamics, and voice-quality measures.

#### 4.3.3 Interpretation of pseudo-label performance

Because the sentiment labels were generated using an SST-2-derived weak-supervision pipeline, classification performance in these experiments should be interpreted as agreement with pseudo-labels rather than as independently validated clinical sentiment accuracy. The Random Forest model was trained to reproduce these pseudo-labels from text and acoustic features; therefore, high agreement with the pseudo-labeling pipeline does not by itself establish validity of affective-tone recognition in aphasic speech. We therefore removed the chi-square agreement analysis and instead report ablation results together with the limitations of the weak-supervision strategy.

### 4.4 Multimodal sentiment classification

The revised multimodal sentiment classification results are reported through the text-only, audio-only, and fused text-audio ablation analysis in Table 4. This table replaces the earlier standalone classification-report figures because it evaluates all feature sets under the same Random Forest framework and includes both utterance-level and recording-disjoint splits.

**Table 4:** Ablation analysis comparing text-only, audio-only, and fused text-audio Random Forest classifiers under utterance-level and recording-disjoint splits.

Split	Feature set	N train	N test	Accuracy	Macro-F1	Negative F1	Positive F1
Utterance-level	Text only	2,173	544	0.967	0.645	0.983	0.308
Utterance-level	Audio only	2,173	544	0.647	0.438	0.781	0.094
Utterance-level	Text+Audio	2,173	544	0.967	0.645	0.983	0.308
Recording-disjoint	Text only	2,186	531	0.979	0.791	0.989	0.593
Recording-disjoint	Audio only	2,186	531	0.554	0.388	0.706	0.071
Recording-disjoint	Text+Audio	2,186	531	0.979	0.791	0.989	0.593

Under the recording-disjoint split, the fused text-audio model did not improve over the text-only model, while the audio-only model performed substantially worse. These findings indicate that the current classification performance is primarily driven by textual embeddings. Therefore, the acoustic component is interpreted cautiously as exploratory rather than as evidence of robust utterance-level affect detection.

### 4.5 Human-centered evaluation (IRR and NDCG)

We evaluated model sentiment predictions through a small manual rating study and ranking-based agreement analysis to examine how model outputs aligned with human perception.

#### 4.5.1 Manual evaluation results and inter-rater agreement

Five independent raters evaluated 35 complete items across three subsets: aphasic utterances, control utterances, and model prediction examples. Ratings were assigned on a 1–4 scale based on the perceived fit between the predicted sentiment and the utterance’s affective tone, conversational context, expressed intensity, and coherence.

Inter-rater agreement varied substantially across subsets. Agreement was lowest for aphasic utterances (Fleiss’ κ=0.053, N=11), indicating minimal agreement beyond chance and highlighting the ambiguity of interpreting affective tone in fragmented aphasic speech. Agreement was higher for control utterances (Fleiss’ κ=0.388, N=13) and moderate-to-low for prediction examples (Fleiss’ κ=0.257, N=11). These results suggest that human interpretation of affective tone is itself uncertain in aphasic discourse; therefore, the manual evaluation should be interpreted as a small feasibility audit rather than definitive validation of model correctness. Table 5 summarizes the human evaluation results by dataset.

**Table 5:** Human evaluation summary by dataset.

Dataset	N items	N raters	Fleiss’ κ	Mean NDCG	SD NDCG	Random NDCG
Aphasia	11	5	0.053	0.967	0.028	0.912±0.046
Control	13	5	0.388	0.946	0.028	0.884±0.055
Predictions	11	5	0.257	0.972	0.013	0.925±0.037

Fleiss’ κ measures inter-rater agreement across four rating categories. NDCG is computed using consensus-normalized item rankings, with a random-ranking baseline shown for interpretation.

**Figure 9:**
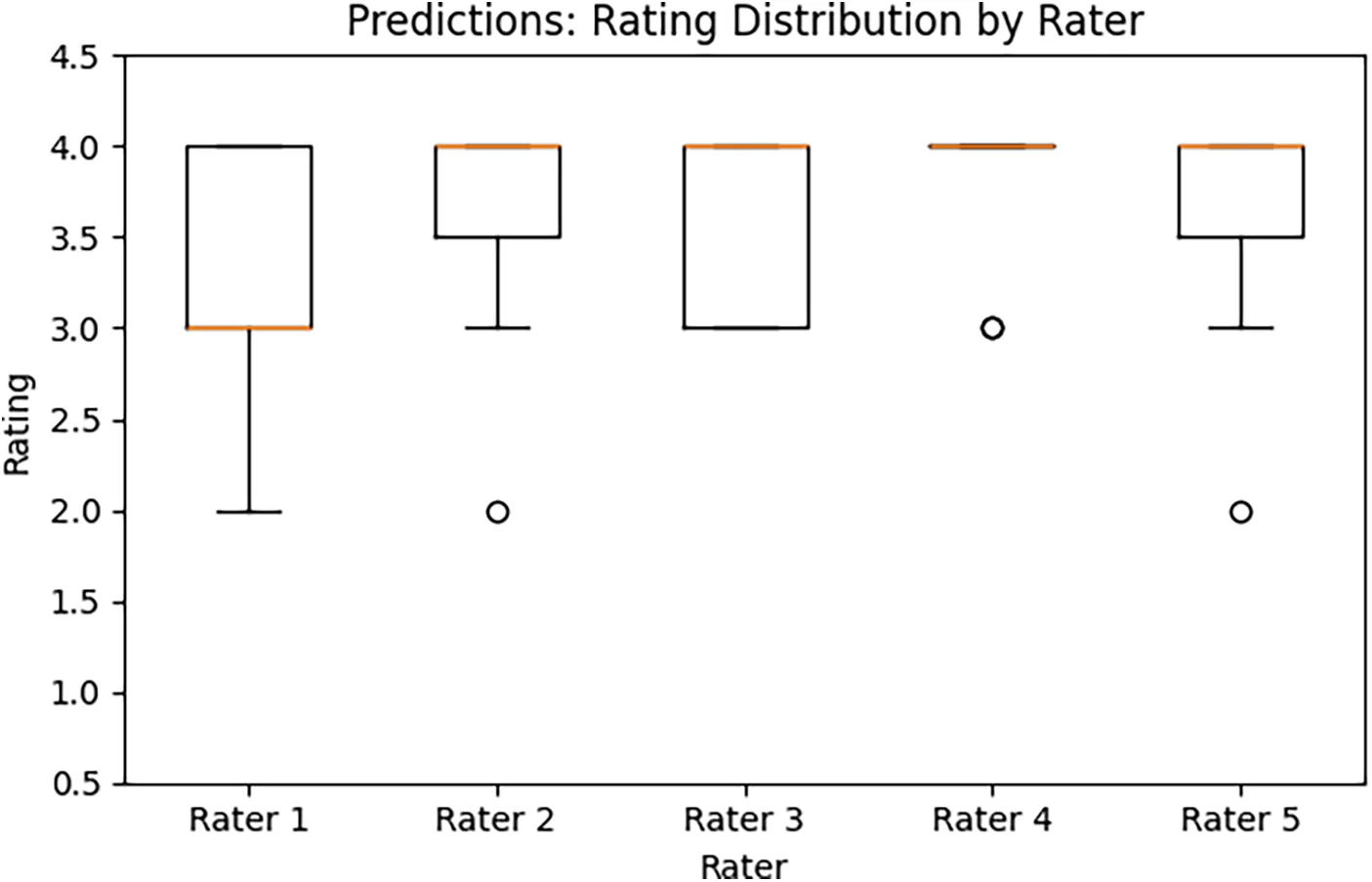
Prediction examples: rating distribution by rater. Ratings are shown on a 1–4 scale and summarize rater-level variation in perceived sentiment fit.

**Figure 10:**
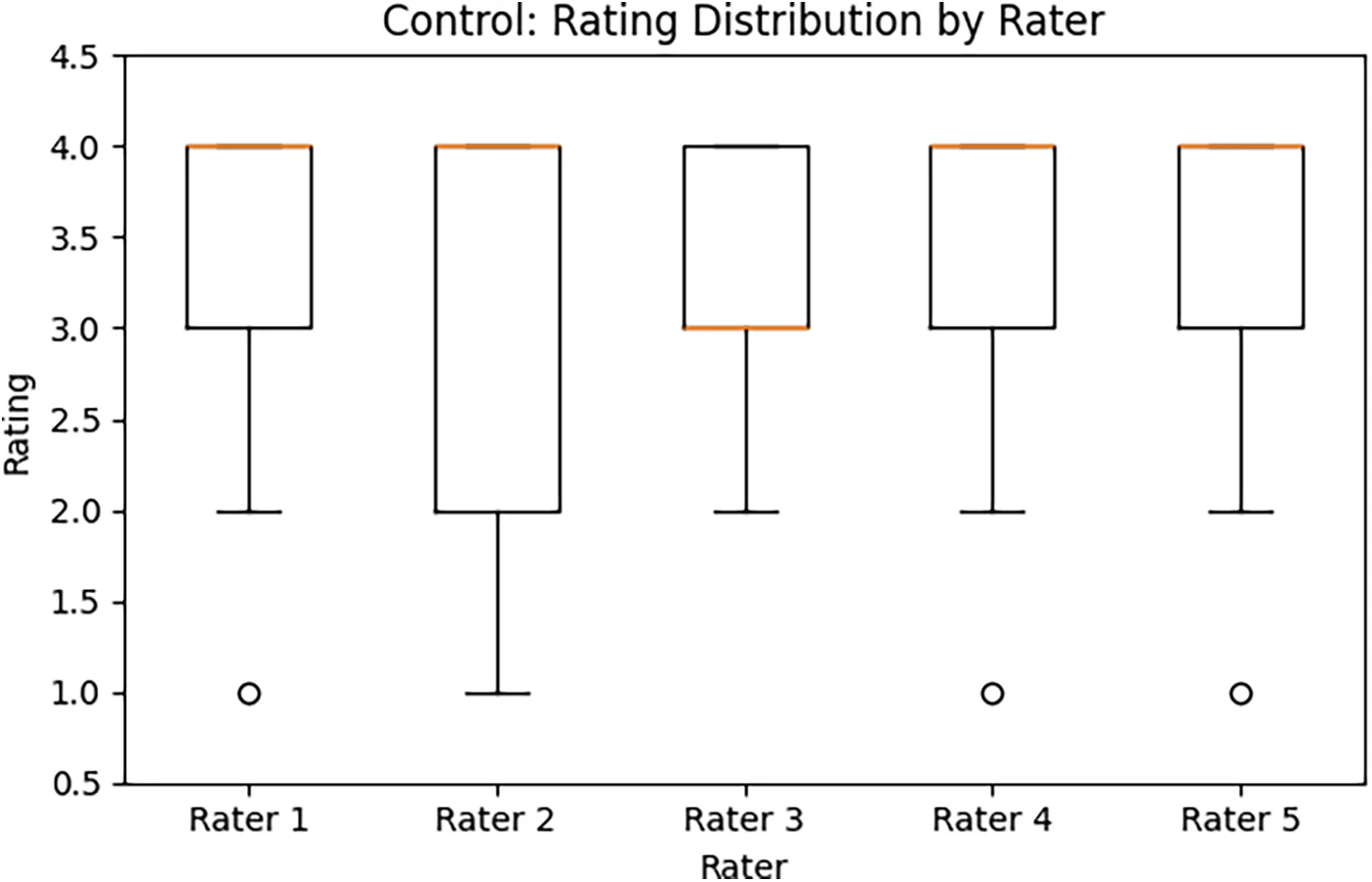
Control subset: rating distribution by rater. Ratings show comparatively higher agreement than the aphasia subset, although agreement remains limited.

**Figure 11:**
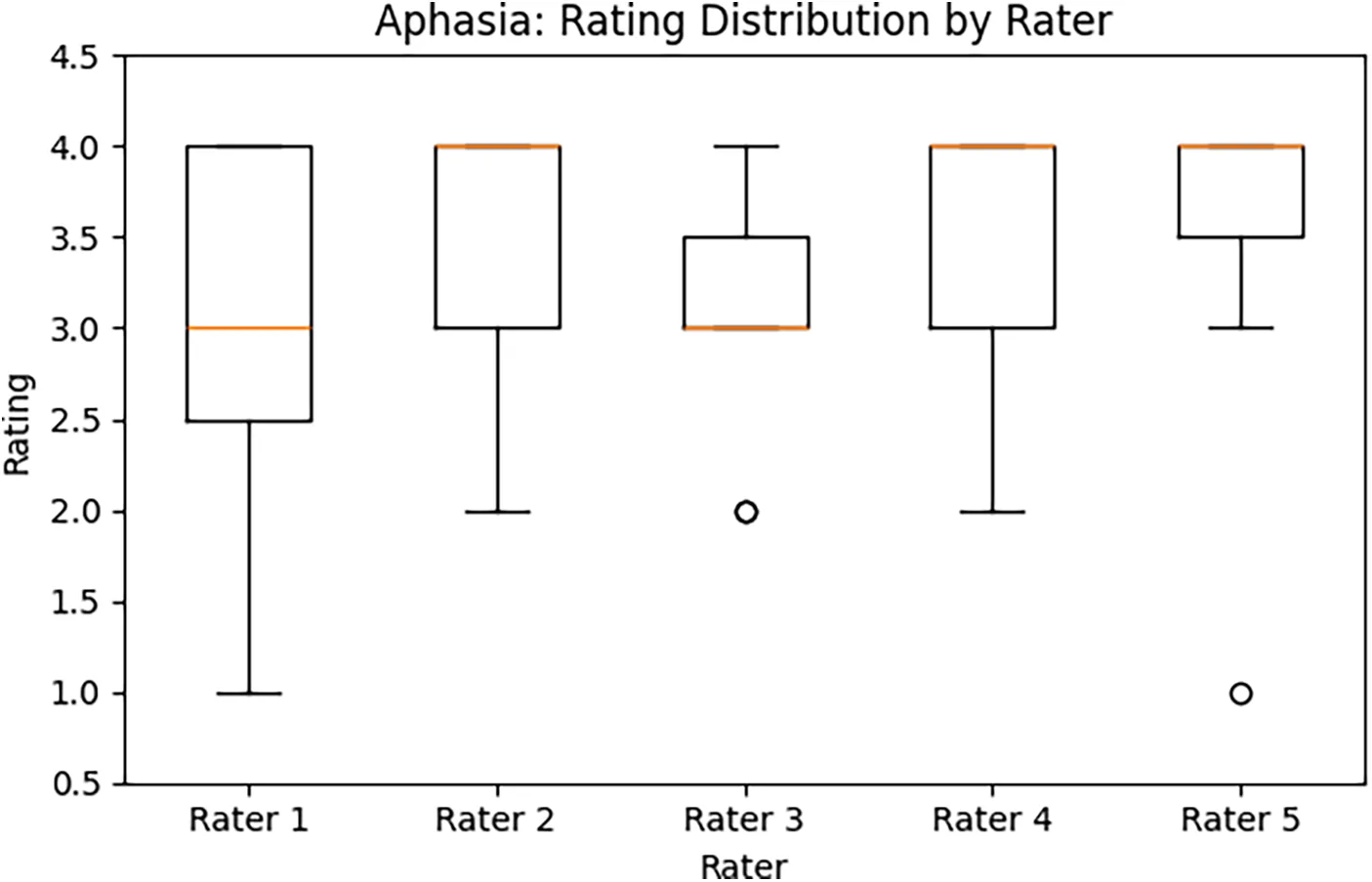
Aphasia subset: rating distribution by rater. Wider variation across raters reflects the ambiguity of affective-tone interpretation in fragmented aphasic speech.

The NDCG values suggest that rater rankings were generally aligned with consensus rankings within each subset. However, because the random-ranking baseline was also relatively high, NDCG should be interpreted as a ranking-alignment measure rather than as evidence of classification correctness. Together, the low Fleiss’ κ for aphasic utterances and the limited sample size emphasize the need for a larger independently labeled clinical evaluation set in future work. Rater-level score distributions for prediction examples, control utterances, and aphasia utterances are shown in Figures 9–11.

## 5 Discussions

### 5.1 Key findings and implications

This study provides an exploratory weakly supervised analysis of affective-tone proxy patterns in AphasiaBank speech transcripts with paired audio. The revised results show that aphasic utterances were more often assigned negative SST-2-derived pseudo-labels than control utterances. Specifically, after filtering to valid binary pseudo-labels, aphasia utterances were 73.7% negative and 26.3% positive, while control utterances were 59.3% negative and 40.7% positive.

These group-level differences should be interpreted cautiously. Because the labels were generated using an SST-2-derived weak-supervision pipeline, they do not represent independently validated emotional states. The observed patterns may reflect affective tone, but they may also reflect differences in language structure, task content, topic, age distribution, sampling, or domain mismatch between SST-2 and clinical aphasia speech.

Age- and gender-stratified summaries were used as descriptive analyses to examine possible subgroup variation in pseudo-label distributions.This cautious interpretation is important because sentiment models can encode demographic and age-related biases, which may distort affective interpretations across speaker groups [42]. These subgroup findings should not be interpreted as validated demographic effects. Instead, they motivate future work using independent human annotation, larger balanced subgroup samples, and stronger controls for task, age, gender, and topic.

### 5.2 Challenges in multimodal integration

The revised ablation analysis showed that the fused text-audio model did not improve over the text-only model under the recording-disjoint split. In contrast, the audio-only model performed substantially worse, especially for the positive class. These results indicate that the current classification performance is primarily driven by textual embeddings rather than acoustic features.

This limitation is likely related to the way the acoustic features were extracted. The available acoustic representation was summarized at the recording level and then attached to multiple utterances from the same recording. As a result, the acoustic features were not truly utterance-aligned and may not capture local affective cues such as pitch variation, intensity dynamics, pauses, voice quality, or prosodic emphasis. Future work should therefore use utterance-aligned acoustic features and stronger audio-text fusion methods before making claims about robust multimodal affect detection in aphasic speech.

### 5.3 Improving model robustness and generalizability

One promising avenue is the implementation of attention mechanisms to better align audio features with their corresponding textual embeddings. Current approaches assume uniform relevance of audio features across sentences, which can lead to suboptimal results. Attention mechanisms can dynamically weigh the importance of specific audio features, ensuring more precise integration with text data. Additionally, temporal segmentation can be employed to match audio features to sentence boundaries more effectively, further improving the synchronization between modalities.

To enhance multimodal feature representation, future work should explore advanced models such as AudioBERT and CLIP. These architectures are designed for cross-modal learning and can capture complex interactions between text and audio data, offering improved performance over traditional models. The use of these models would allow for deeper insights into how linguistic and acoustic features interact in aphasic speech, providing a more nuanced understanding of the emotional landscape.

Demographic-aware sentiment classification presents another critical area for exploration. Fine-tuning transformer models, such as BERT, with demographic embeddings (e.g., age and gender) alongside text embeddings could provide more personalized analyses. Incorporating these embeddings would allow models to account for demographic-specific linguistic and emotional variations. Furthermore, employing multi-task learning frameworks that simultaneously predict demographic attributes and sentiment could lead to more robust and interpretable models.

### 5.4 Addressing sentiment imbalance with data augmentation

Another area of improvement lies in the augmentation of datasets to address class imbalances and the sparsity of positive sentiment examples. Synthetic data generation techniques, such as oversampling and adversarial data synthesis, could be utilized to create a more balanced dataset, allowing models to learn a more representative distribution of sentiments.

### 5.5 Expanding the scope of multimodal sentiment analysis

Finally, expanding the scope of analysis to include cross-linguistic datasets and diverse cultural contexts could generalize the findings across broader populations. This expansion would require adapting models to account for language-specific features and cultural nuances, presenting a new frontier in multimodal sentiment analysis.

By addressing these directions, future research can pave the way for more accurate and adaptable multimodal systems. These systems may support future research aimed at improving the understanding and management of aphasia and other communication disorders, with the long-term goal of improving quality of life for affected individuals.

## 6 Conclusions

This study presented an exploratory weakly supervised analysis of affective-tone proxies in AphasiaBank speech transcripts with paired audio. DistilBERT-derived text embeddings and recording-level acoustic summaries were evaluated using Random Forest classifiers under utterance-level and recording-disjoint splits. The revised ablation analysis showed that text-only and fused text-audio models performed identically under the recording-disjoint split, while the audio-only model performed substantially worse. These findings indicate that the current classification performance is primarily driven by textual embeddings, and that the recording-level acoustic features should be interpreted as exploratory rather than as evidence of robust multimodal affect detection.

Across the pseudo-labeled corpus, aphasic utterances were more often assigned negative pseudo-labels than control utterances. Age-stratified summaries showed subgroup variation, but these patterns should be interpreted descriptively because they are based on model-derived pseudo-labels rather than independently validated clinical sentiment labels. However, because the labels were generated through an SST-2-derived weak-supervision pipeline, these results should be interpreted as affective-tone proxy patterns rather than clinically validated sentiment labels. The human evaluation further showed low inter-rater agreement for aphasic utterances, emphasizing the ambiguity of affective interpretation in fragmented clinical speech. Future work should use independent clinical annotation, utterance-aligned acoustic features, and stronger controls for age, task, topic, and domain bias.

## Data Availability

The datasets analyzed in this study are not publicly available. Access to the AphasiaBank corpus is restricted and requires registration and a signed data use agreement through TalkBank/AphasiaBank. Researchers interested in accessing the data may submit a request at https://talkbank.org/aphasia/.
